# A Chronicle on Genetics and the March of Darwinian and Mendelian Medicine

**DOI:** 10.1186/s12052-020-00129-5

**Published:** 2020-07-02

**Authors:** Diddahally R. Govindaraju

**Affiliations:** Institute for Aging Research, Albert Einstein College of Medicine, 1300 Morris Park Avenue, Bronx, NY 10461 USA

**Keywords:** Ken Burns, Siddhartha Mukherjee, Garrod, Genetic disorders, Huntington disease, Precision medicine, Genome editing, Genetic medicine

## Abstract

Ken Burns, a well-known documentarian has produced a film titled, “The Gene: An Intimate History” based on Siddhartha Mukherjee’s book by the same name. The film focuses sharply on the history of genetics, the science of inheritance and variation, in particular, the gene, the central player in genetics, its discovery, structure and function as well as its myriad influence on development, growth, health, disease and evolution of all organisms. Importantly, he has shown how geneticists, physicians, patients and their families as well as society at large must cooperate, develop mutual trust and share ethical responsibilities in order to predict, prevent, manage and cure many inherited disorders. Mr. Burns has reinforced these ideas beautifully with illustrations, archived film footage, location filming and commentaries. The film also hints at the need for taking a unified Darwinian-Mendelian and Hippocratic approach toward building a healthier human society. In short, this is a beautiful and engrossing documentary on the future of Genetic Medicine.

## Details of the documentary film

Burns, K. The Gene: An intimate history presented in two parts on April 7, and April 14, 2020 on PBS.

“As to what constitutes fitness to survive, man and Nature do not see eye-to-eye… The whole aim of medical art, whether therapeutic or preventive, has been to counteract the laws of Nature”-Archibald Garrod (1931).

A two-part documentary film titled, “The Gene: An Intimate History” by Ken Burns, a celebrated documentarian, was aired on PBS on April 7, and April 14th. The film is an adaptation from a book with the same title by Siddhartha Mukherjee (2016). The first and the second part of the documentary deal with respectively: (a) dawn of the modern age of genetics, and (b) revolution in the treatment of (genetic) disease. In the documentary “avatar” of Mukherjee’s book, Mr. Burns traces the antecedent roots of genetics and articulates the contemporary euphoria about genetic research, toward predicting, preventing and potentially curing inherited disorders, being popularized as precision medicine. His narrative sharply focuses on the common thread shared among genes in the human genome, their structure, the vision to map and sequence them as well as the opportunities genetic research offers for alleviating human suffering from diseases of genetic origin. The focused approach makes the documentary more engaging relative to the book. The objectives of this review are to: (a) briefly introduce the scope of the original book source, (b) discuss the history of genetics and its application to human health as featured in the documentary, and (c) reflect on the emerging trends toward integrating the Darwininan and Mendelian principles with other advances in modern medicine, and the plausible impact of such an alliance on future health care.

In his book, Mukherjee first reflects on the inheritance and distribution of mental illness among his family members as a prelude to describing the discovery of the laws of inheritance and variation among a set of crosses of garden pea varieties and their progenies by Gregor Mendel in 1865 within the confines of a “Walled Garden” in Brno, Czechoslovakia. In the same period, Mendel’s contemporaries, Darwin and Wallace successfully advanced the Theory of Natural Selection. While Darwin was inspired by variation exploited by plant and animal breeders, Mendel meticulously recorded and analyzed the observed the pattern of variation among his crossing experiments involving specific parental lines, their hybrids and the progeny of those hybrids. Note that Darwin also carried out extensive studies on the effects of cross and self-fertilization in plants. He notably struggled to provide a tangible explanation for the inheritance and distribution of variation (both in nature and among his crossing experiments), which provides the raw material for all evolutionary processes. Darwin was, however, cognizant of the health consequences of inherited variation, especially consanguinity (inbreeding), which took a toll on the health of his children and grandchildren owing to his marriage to his first cousin. Indeed, health and disease are inextricable aspects of evolutionary process (Haldane 1949). The Mendelian principles rediscovered 120 years ago, were quickly extended to study human health and disease (Garrod 1902). Subsequent application of both Darwinian and Mendelian principles to crop and animal breeding has contributed greatly toward ameliorating world’s food scarcity. Could these discoveries be employed to cure diseases and manage human health?

Despite a few major omissions and a clear bias toward molecular biology (Govindaraju 2016), Siddhartha Mukherjee’s historical narrative of the science of genetics or heredity and variation remains as an important guide to study the origin and growth of genetics, and its relevance to modern medicine. It also mirrors the abuses of genetic discoveries to propagate racism, the fallacy of biological determinism and warns about the consequences of tinkering the human genome with bad intentions, which could seriously impede the progress of not only genetics and evolutionary biology, but also the entire human society. He has also discussed that genetic discoveries made using the same principles that Nature has tinkered with, over billions of years, could be employed to managing and curing a number of diseases. Such changes, prudent or imprudent, could also chart novel trajectories of human evolution.

Ken Burns, like other great film directors, has taken only the scaffoldings from the original source, and has retold the history of genetics in this documentary from an astonishingly fresh perspective. In a way, it feels like watching a new and independent film! The documentary is rich with appropriate illustrations and commentaries, photographs from archives, filming on actual locations and footage showing the interaction of physicians and scientists with children and their parents, and patients and their families. These scenarios seamlessly intercalate with each other and provide a rich tapestry of the science of genetics (and its evolutionary roots and trajectories) and what it could offer to improve human health in the years ahead. Mr. Burns focuses sharply on genes influencing human traits and specific inherited disorders, their structure and organization, and illustrates with the aid of case studies how to predict, prevent and possibly cure genetic disorders using genomic information.

The film opens with the presentation of a bold, but professionally and socially irresponsible gene editing experiment conducted by a Chinese scientist, Dr. He Jiankui. He used a novel genome editing technique called “CRISPR-Cas9” (clustered regularly interspersed short palindromic repeat) system on human embryos (germ-line editing) in order to cure twin girls from HIV. The embryos were developed from in vitro fertilization involving HIV positive father and HIV negative mother. Note that individuals who are heterozygous to “Delta 32” mutation in the *CCR5* receptor gene are susceptible to HIV, but homozygous ones show greater levels of resistance. Germ line editing is irresponsible because the genetic modifications to the developing embryos are inherited and their life-course consequences on development and health as well as evolution have not been thoroughly investigated by a panel of international body of responsible citizen scientists. Mutations at the *CCR5*—Delta 32 site occurred many generations ago in Europe and spread in human populations, obviously much before the appearance of HIV.

Pediatric geneticist Dr. Wendy Chung introduces us to rare childhood diseases, which follow a Mendelian pattern of inheritance. The introduction is facilitated through the anxious and devoted couple, Luke and Sally Jackson, and their beautiful young child Susanna. Unfortunately, Susanna carries a defective (mutant) in “*KiF1A*” gene, which leads to a degenerative neural disease, “Hereditary Spastic Paraparesis.” The disease would not only take away her childhood but also eats away her brain bit-by-bit over time. Dr. Chung patiently plays with the child and observes her subtle movements for diagnosing the disease progression. Here we glimpse not only the dedication of Susanna’s parents to discovering the cause of their daughter’s illness, but also of Dr. Chung’s (and many physicians like her) palpable devotion to her patients in order to alleviate their suffering and find a purpose in her profession and life. The consequences of the mutation on protein and disruption of cellular function and its eventual manifestation as a disease are beautifully illustrated.

As a rule, mutations exert a broad spectrum of adverse, cumulative, pleiotropic and contextual effects on the expression of a given trait or a disease; but a fraction of them contribute to good health as well. Both advantageous and harmful mutations are nonetheless maintained among individuals and populations of all life forms including humans in accordance with Darwinian principles. This prompts us to ask enduring questions on the nature of human existence, inheritance and variation, as well as human destiny. Greek philosophers and thinkers such as Pythagoras, Aristotle and Hippocrates also pondered these questions and speculated wildly on the birth of offspring, treating diseases and restoring human health.

Population growth and the demand for food over the centuries in Europe needed new innovations in food production. Gregor Mendel, being the son of a farmer himself, sought to discover the principles of inheritance, on the insistence of F. C. Napp—“what is inherited and how?” (Henig 2000) with the ultimate goal to increase the food production and expand economic growth in the province of 19th century Moravia, based on scientific principles. Mendel’s experiments on the pattern of inheritance and distribution of “recessive and dominant” traits among the progeny derived from crosses of varieties of peas at his garden in Brno are beautifully presented in the film with graphics and commentaries. Mendel’s experimental results, however, did not “basically vanish” from the scientific community (for 35 years) but were ignored by his more influential colleagues such as Carl Nageli and others (Olby 1966). Apparently, this unpretentious Monk was fond of saying to his friends, “*Meine Zeit wird schon kommen*”–“My time will come” (Henig 2000). Indeed, sometimes, truth takes longer time to establish itself!

Following the rediscovery of Mendelian Laws of hereditary “Merkmals/factors” (later termed genes), their alignments like beads on a string of chromosomes and their variation were investigated (among others) primarily by T.H. Morgan and his students at Columbia University’s “Fly Room.” This led to discovering the origin of new mutations that generate heritable variations is presented beautifully with illustrations. On the other hand, some other scientists, largely influenced by the Galton School were advocating “genetic determinism.” A band of international scientists led an organized and often militant propaganda for applying Darwinian-Mendelian principles to “re-engineer” human society by sterilizing “feeble minded” and “unfit” individuals carrying undesirable traits, in the name of eugenics. The eugenics movement is very well brought out in the film using conditions that prevailed in both Hitler’s Germany and the treatment of mental patients in the United States.

What is the nature of hereditary material? It must be a chemical. This idea is attributed to Irwin Schrodinger from his popular book “What is Life” published in 1944. However, Oswald Avery also published his results that genes and chromosomes are composed of DNA in the same year. But the original insight on DNA (nuclein) as the material responsible for inheritance came from Friedrich Miescher in 1871, only 6 years after Mendel’s publication. I wish the commentators mentioned Meischer’s contribution to the chemical nature of hereditary material—DNA. Interestingly, the discoveries by Darwin-Wallace, Mendel, and Meischer in a span of about 11 years suggests the intense interest that was prevailing in mid-19th century Europe to understand the nature of inheritance and variation.

Following Morgan’s pioneering attempt to map genes on chromosomes like beads on a string using the fruit fly as the model organism, the next major challenge remained–understanding the chemical structure of the DNA molecule. Two very talented and driven scientists, James Watson and Francis Crick meet at Cambridge University to take up this challenge. Both Watson’s and Crick’s drive, complementary interests, intuitions, open-minded trans-disciplinary thinking and intuitive grasping of the double-helical structure of the DNA molecule by Watson, their collaboration, discussions, building models, interaction with Maurice Wilkins and learning from Erwin Chargaff on nucleotide pairing are well presented. Watson’s peek view of the X-ray picture of the DNA molecule prepared by Rosalind Franklin, during his discussions with Wilkins, and his conviction that DNA molecule must have a double-helical structure reminds me of Louis Pasteur’s dictum “Chance favors the prepared mind.”

Subsequent to the discovery of DNA structure, details about the components of the central dogma in molecular biology (DNA codes for RNA which in turn guides protein synthesis) emerged and its details are beautifully presented. Also, new techniques were developed to mapping genes based on their degree of correlation with the neighboring genes using DNA markers. In this period, a woman named Leonore Wexler was diagnosed with Huntington disease (HD), an insidious and devastating neurological condition, which shows a dominant pattern of Mendelian inheritance. Her daughter, Nancy Wexler, although lacking formal training in genetics takes up the challenge to discover the genetic basis for HD. This requires collecting information on individuals nested in families who carry this disease. Nancy Wexler discovers that such families live in the village of Barranquitas near Lake Maracaibo, Venezuela. This population is derived from a few founding families (founder population) settled in this area in the early 19th century. The mutation that influences HD came with the founding members. Over the years, it has gradually spread among the present-day inhabitants of the Barranquitas village. How did the disease go unnoticed by the community for so long? Sadly, the disease is an insidious imposter; it is generally expressed in seemingly healthy individuals only after their prime reproductive age. By then, most couple would bear healthy looking children, but the gene responsible for the disease (*HTT*) would be concealed in some of these individuals and work stealthily and steadily till they pass their reproductive age. This hide-and-seek pattern of the disease spread continues with the expansion of the community. Ms. Wexler’s compassion, understanding, and interaction with the affected individuals among families in this community, meticulous note-taking, and pedigree construction sets a great example for geneticists and genetic counselors. The scene in which Dr. Wexler affectionately picks up and kisses a boy with HD is one of the most poignant pieces of footage in the film and perhaps in all of medical/human genetics. It shows the passion and dedication these investigators have toward their study subjects, who cry for help and need relief from their diseases. The collection of blood samples from affected pedigrees led to the identification of the *HD/HTT* gene and the excess of CAG nucleotides repeats in the HD gene among the HD patients. Could the knowledge gained on HD on the Venezuelan HD patients be used on others living elsewhere afflicted by the same disease? Yes, indeed! A number of individuals in Peter Allen’s family in Chelmsford, Essex, U.K., including his mother and grandmother died from this disease. Mr. Allen is now showing signs of the disease; his siblings could be carrying this gene as well. Their mutual struggles and support as well as their optimism are well documented.

Advances in DNA technology heralded by many molecular biologists prompted them to create novel variation as it is done naturally among organisms by a process called recombination, but only at an unprecedented speed in Petri dishes. Paul Berg, in particular, attempts to repeat an evolutionary process under laboratory conditions that bacteria have evolved over time. Using the same mechanisms used by bacteria in nature, he engineered “recombinant DNA” in plasmids and re-introduced them into bacteria. This discovery spawned a new discipline called “Genetic Engineering.” This also led to many demonstrations and public discourse against recombinant DNA research. These experiments have progressed non-stop following the establishment of ethical standards, and have transformed the face of biological and medical research.

In the second part of the documentary titled, “Revolution in the treatment of (genetic) disease” the director focuses on the advances made toward understanding the genetic bases of diseases and how we could harness the emerging ideas in molecular biology to treat human diseases. Why are certain genes expressed at certain stage in life or in a specific environment and stop functioning at a different stage in life or under different environmental conditions? Simply, how are genes regulated? Francois Jacob and Jacques Monod in France provided a convincing answer to this question known as operon theory. I only wish the documentary had mentioned Barbara McClintock’s “controlling elements” work, which in principle, is similar to Jacob and Monod’s study, but preceded their work by over a decade!

Time was ripe for asking an audacious question. Could genetic discoveries made on model organisms to understand molecular mechanisms and to manipulate DNA in microorganisms be employed to cure or manage human health for the benefit of humankind? If so, could we sequence the entire genome to both count and chart the detailed organization of genes in the human genome? This grand question led to the launching of the “Human Genome Project” (HGP) in 1993. The race to read the entire human genome, letter-by-letter culminated in 2003, through “soft power” diplomacy and the announcement of the feat by the then President of the United States, Bill Clinton, is well presented. Two decades since the completion of the HGP, public awareness of genes and genetics and their role in health and disease has become as common as the Laws of Gravitation or Theory of Relativity in everyday parlance.

Humans are affected by both common genetic disorders such as Type 2 diabetes, heart diseases, asthma, schizophrenia, etc. and nearly 7000 rare genetic (Mendelian) disorders. The genetic architecture of many common genetic disorders is more complicated than the genetic bases of Mendelian disorders. While the HGP was progressing, geneticists contemplated on understanding both the genetic basis of human disorders and curing some of them using recombinant DNA techniques. In a bold attempt to cure a classic Mendelian disorder, ornithine transcarbamylase (OTC) deficiency in a young patient, James Wilson attempted to use genetically engineered viral particles at The University of Pennsylvania. Unfortunately, the patient died of complications on Sept 17, 1999. The study was also an ethical and a scientific quagmire and the hope for “gene therapy all but died with him.”

Yet, in the last two decades many new molecular approaches to manage or cure genetic disorders have been developed. One of the most promising and sought after technique is the CRISPR, discovered by Dr. Jennifer Doudna and Dr. Emmanuelle Charpentier. The technique exploits a trick that bacteria have evolved in order to recognize and kill invading viruses. Note that the same approach was exploited by Dr. He Jiankui for editing human germ cells. In addition, bright and bold individuals such as the Rosens, Wexlers and Allens, although not trained in genetics or medicine, are strengthening the hands of dedicated physicians such as Dr. Wendy Chung toward diagnosing and treating patients with genetic disorders. On the other hand, some of these patients, their families and support groups are also striving equally hard to understand the genetic bases of the diseases they are carrying so that they could manage their own diseases slightly better and lead a relatively healthy life. They are also joined by scores of laboratory scientists, their technical and nursing staff as well as the dedicated members of society around the globe. The global community of collaborators generate public interest on specific genetic disorders and build disease specific networks, in order to alleviate the suffering (and stigma) inflicted by deleterious mutations among genes on the individuals and families that carry them.

Take for instance, Ms. Audrey Winkelsas, a young and bright graduate student working at the NIH and Oxford, is suffering from Spinal Muscular Atrophy (SMA). The disease gradually attenuates muscle strength, but the rate of its progression varies among individual patients. She is determined to find the molecular bases of the cause and a potential cure or at least to stall its progression. She has taught her mother to perform the required laboratory analyses, while the clinical team monitors her progress. Audrey’s physician’s confession about his evaluation of her disease reminds us of the old adage, “An old patient is better than a new doctor.” Similarly, Mr. and Mrs. Jeremy and Cheryl Yoder have their daughter Ariel. The child is severely affected by SMA, but her disease has progressed to the point of no return. Cheryl gives birth to another child. Unfortunately, the new baby also has SMA! Their pediatrician suggests that they could try an experimental drug if they could bring the child for treatment before the disease is overtly expressed. The timing of diagnosis is critical if some of these therapies to be effective. Another patient, Ms. Lessie Lassiter has breast cancer, which is a complex evolutionary disease. She receives an experimental drug, which is improving her situation. Ms. Lassiter is happy and hopes she would lead a normal healthy life like the rest of us! Her case serves as a beacon of hope for all breast cancer patients that the culprit mutation(s) could be identified and treated with precision with little or no side effects. On a similar note, Mr. David Williams is suffering from sickle cell disease, which affects nearly 250 million children worldwide. A new technique involving correcting the mutation that causes red blood cells to assume a crescent shape is used to re-engineer stem cells harvested from Williams’ own body. The newly “engineered” stem cells are transfused into Williams’ body using the standard medical procedures. With just one such “somatic cell therapy” treatment, he expects to lead a relatively long and healthy life.

Application of new molecular techniques has paved the way for understanding the nature of human diversity and the prevalence of diseases including Mendelian disorders. But, approaches to decipher the genetic bases of most complex diseases, have worked “less well than hoped for,” also implies that gene centric approaches may not offer a general panacea to treat all genetic disorders.

Nonetheless, by applying new discoveries made in genetic medicine, Ms. Winklesas’ grip strength has improved. The Yoder’s second child walks across the room into her parents’ open arms, and walks next to him, he carries the child on his shoulders. Their jubilation is priceless. Mr. Williams is energized and his mother is happy. Mr. Allen, out in the UK, receives “genetic medicine” contained in a “small vial of hope.” As a result, Mr. Allen, who couldn’t draw a shape or write clearly, could do both reasonably well. His satisfied wife watches his progress with a sigh of relief. We come to appreciate that lessons learned by understanding the origin and evolution of cancer are also identical to evolutionary mechanisms common to all organisms, including humans. In fact, cancer has been recognized as an evolutionary disease. Therefore, application of Darwinian and Mendelian principles together with classical and medical treatments has become integral part of clinical care and management of cancer patients (Swanson 2020). Such nexus approaches, now armed with rapidly evolving genome editing (both germ-line and somatic line) techniques, could change the future course of not only cancer treatment, but also diseases caused by single or only a few base pair changes, such as progeria, sickle cell anemia, hemophilia, cystic fibrosis, deafness, blindness and hundreds of similar genetic conditions (Rosen 2020).

The scenes showing helpless innocent looks of children and the faces of anxious and tired parents of patients are very touching. These images haunt us all. Watching the agony of innocent children, their parents and caregivers reminds us of the Buddha’s counsel to Kisa Gotami that their losses and the pain they endure are ours as well.

In short, Ken Burns and his team deserve accolades for making this beautiful documentary on the convergence of genetics, evolution, and the future of genetic medicine (Childs 1999). This film reminds me of another beautiful documentary, “Ascent of Man,” by the late Jacob Bronowski on the history of science aired over 40 years ago on PBS. Mr. Burns has gradually and meticulously shown the need for understanding genetic variation in order to chart new territories in the future medical practice ranging from cancer to COVID-19, recently touted as “Precision Medicine.” In doing so, he and his team have emphasized the ethical and moral responsibilities human society as a whole has to share and shoulder in order to fully harness the Promethean power of evolution and genetics which could potentially and perhaps permanently transform human micro-evolutionary processes as well as future health care. An old saying goes, that “A picture is worth thousand words.” If so, this films speaks volumes on the place of genetics and evolution as well as the challenges that lie ahead in the practice of future medicine. This film deserves to be watched not only by geneticists, evolutionary biologists, and medical professionals but also anyone who wants to know who we are? Where did we come from? Why we look the way we do? Why we get certain diseases, why some of us live longer than others—that means each and every one of us! The Gordian knot between evolution and medicine recognized by Garrod (1931), the father of precision medicine (Perlman and Govindaraju 2016) nearly a century ago is being gradually disentangled with Darwinian and Mendelian mechanisms themselves. The film clearly demonstrates the transformative power of the confluence among Darwinian, Mendelian and Hippocratic medicine as well as the shared moral and ethical responsibilities of geneticists and physicians toward building a healthier human society.

## Data Availability

Because this article is classified as a Book Review, No datasets were used or generated and analysed for writing this article.
